# Toward better data disaggregation: A person-centered approach to understanding AANHPI sociodemographic diversity in resource constrained times

**DOI:** 10.1371/journal.pone.0336912

**Published:** 2025-11-19

**Authors:** Lu Dong, Jaimie Shaff, Douglas Yeung, Ruolin Lu, Delia Bugliari, Anthony Rodriguez, Anita Chandra

**Affiliations:** 1 RAND, Santa Monica, California, United States of America; 2 Department of Psychology, Stony Brook University, Stony Brook, New York, United States of America; 3 RAND, Arlington, Virginia, United States of America; 4 RAND, Boston, United States of America; Virginia Commonwealth University School of Medicine, UNITED STATES OF AMERICA

## Abstract

**Background:**

Each year, the United States loses billions of dollars due to health inequities. Data disaggregation is essential for understanding the health status and needs of populations to identify these inequities and inform efficient resource allocation. For example, aggregating data from people identifying with Asian, Native Hawaiian, and other Pacific Islander (AANHPI) communities may inhibit the identification of important health challenges within this large and diverse community, impeding meaningful progress toward reducing differences in health outcomes.

**Methods:**

This study employed Latent Class Analysis (LCA) to identify meaningful subgroups within the AANHPI population. Two studies were conducted: Study 1 analyzed data from the Amplify AAPI Survey, which included 1,026 AANHPI adults, while Study 2 utilized the 2023 National Survey of Health Attitudes (NSHA) with a sample of 318 AANHPI respondents. Both studies collected comprehensive sociodemographic measures, including educational attainment, household income, and employment status.

**Results:**

Study 1 identified four latent classes, revealing heterogeneity within the AANHPI sample based on income, education, language use, and generational status. Class characteristics highlighted variations in age, marital status, and employment. Study 2 identified two classes: high socioeconomic status (SES) and low SES. Class characteristics demonstrated differences in age distribution, homeownership, and perceptions of community well-being.

**Conclusion:**

This study demonstrated the feasibility and utility of a person-centered analytic approach like LCA to identify meaningful subgroups within an aggregated population. These findings join a growing body of evidence that emphasizes the complexity within the AANHPI population and the importance of data disaggregation in public health. These insights are crucial for informing targeted interventions and optimizing resource allocation to effectively address health disparities.

## Introduction

Health inequities cost the United States (U.S.) billions of dollars each year [1]. Data disaggregation, or the process of breaking data into subgroups, is a crucial public health activity that can inform actions to meaningfully address these inequities and reduce the economic burden associated with them [2]. In the U.S., disaggregated data may be used to understand the characteristics or needs of different populations, determine how to most efficiently allocate resources, and identify opportunities for tailored efforts.

Data disaggregation is used across industries to allocate resources, from determining how to best invest advertising dollars to deploying limited public health resources. Unfortunately, aging systems and outdated practices can limit efficient resource allocation by concealing populations with the greatest needs. Aging data infrastructure (i.e., inability to ingest disaggregated data [3]), limiting statistical norms (i.e., suppression rules [4]), mischaracterization of populations as “hard to measure” (i.e., limited inclusion of low-income communities in survey data [5]), and concerns about the validity of typical approaches (i.e., perceived unmet need [6]), which meaningfully influence real-world implementation of recommendations and best practices. These structural limitations impede the research community from identifying opportunities to better allocate limited resources.

Public health data are typically disaggregated in alignment with federal national data standards, norms within the field, and local demographics. Within public health, data are disaggregated to understand the health of different populations, identify opportunities to address potential health inequities, and direct limited resources to address the most pressing needs. Throughout this paper, we will be using Asian, Native Hawaiian, and other Pacific Islander populations as an illustrative example. The federal national data standards include two distinct categories for “Asian” and “Native Hawaiian or Other Pacific Islander” [7]. Government classifications have long grouped these populations into a single category, referred to in different ways, such as “Asian American and Pacific Islander,” and public health initiatives are often developed for this aggregate group. This approach to aggregating groups together is often done to simplify political and public sector efforts; additionally, Asian, Native Hawaiian, and other Pacific Islander (AANHPI) communities have a long history of working together to form coalitions and uplift the needs of their communities as part of a larger voice [8,9].

AANHPI describes culturally, linguistically, and ethnically diverse people with origins from over 50 countries and territories [10]. Historical immigration laws, global events, and migration patterns have resulted in geographic diversity of AANHPI heritage groups across the U.S. and will continue to do so as the AANHPI population grows [11]. Accordingly, the need for data disaggregation within the AANHPI population has been well established and can greatly improve the efficiency of resource allocation [12–14]. Subgrouping recommendations by ancestral heritage have been developed and adopted in the 2020 Census [15] and for health survey data in California [16] and New York [12]. However, questions remain on if grouping by ancestral origin is appropriate for identifying health inequities, or if other factors are more salient [17,18]. Additionally, data disaggregation efforts are limited by the ways in which data are collected: many health, state, and local agencies continue to only collect aggregated AANHPI data [19].

Like any other community, different dimensions of wellbeing of AANHPI community members are influenced by social factors such as ancestry group, language, immigration status, and geography [12–14]. Historical events, such as selective immigration policies, influenced the movement of different ancestry groups, positionality in society, biases faced in the U.S., and access to health services [12,14]. Cultural norms, linguistic differences, acculturation, and racialization by phenotypes influence health, perceptions of health status and needs, and multiple steps of the care-seeking process [12,14,20]. These factors impact experiences in the U.S.; the way data are provided, collected, and interpreted; the way interventions are funded, designed, and evaluated; and, ultimately, our ability to make meaningful progress towards all members of our communities experiencing optimal health and wellbeing [21].

Decades of effort have been expended in both advocating and developing methods to facilitate more granular analyses of population subgroups. Many such efforts have focused on increasing data granularity [22], which in turn increases the need to collect greater amounts of data. However, existing data disaggregation approaches may not always be sufficient or feasible, as they fail to capture the important intersectional factors that drive disparities in health outcomes. Methodological characteristics of these approaches may constrain their usefulness. Disaggregation efforts that attempt to disaggregate beyond conventional groups can be impeded by factors such as the way data were collected, lack of statistical power, and risk of identifiability of individuals [22–24]. Further, shifting funding priorities may mean that certain types of data (e.g., race/ethnicity) either cannot be collected, or if already collected, are no longer available [19]. If such real-world policy leads data on race/ethnicity becoming either less available or acceptable, the public health community may need to identify alternate approaches to inform efficient and effective allocation of limited resources. Addressing such concerns may require multi-pronged data disaggregation approaches [25].

Intersectionality research leverages a person-centered theoretical framework with quantitative or empirical methods to incorporate social experiences to develop meaningful subgroups [26]. Data-driven quantitative methods, such as latent class analysis (LCA), have successfully supported exploration beyond variable-centered approaches typically used for identifying opportunities to address health needs [26]. Given limitations of existing data, public health teams working to identify and address health inequities utilize more advanced methods, such as LCA, to better understand and respond to the health needs of their communities that have been masked by less advanced techniques.

The goal of this analysis is to empirically identify and describe distinct sociodemographic subgroups within the AANHPI population using a person-centered analytic approach. Given the considerable heterogeneity in educational attainment, income, language use, and generational status among AANHPI individuals [10], we used LCA to uncover meaningful subtypes based on these core indicators. We then characterized these latent classes using additional demographic variables, including age, AANHPI origin, household size, marital status, and employment status. Finally, we assessed the replicability of the identified class structure using a second independent survey sample. This multi-step analytic approach with the use of two study samples allows for a more nuanced understanding of heterogeneity within AANHPI communities and lays the groundwork for more targeted and data-informed research, practice, and decision-making.

## Study 1: Analysis of the Amplify AAPI Survey

### Methods

#### Participants and procedures.

In a joint effort by RAND and the Robert Wood Johnson Foundation (RWJF), researchers supplemented the 2023 sample for the National Survey of Health Attitudes (NSHA), [27] with additional AAPI respondents drawn from the Amplify Panel, a probability-based panel of AANHPI populations conducted by NORC at the University of Chicago. Data were accessed May-June 2025; authors had no access to information that could identify individual participants during or after data collection.

Participants for the current study were drawn from the May 2024 wave of the Amplify AAPI Omnibus Survey, a national, probability-based survey of Asian American, Native Hawaiian, and Pacific Islander (AANHPI) adults administered by NORC at the University of Chicago. The survey targeted individuals aged 18 years or older residing in the United States, including all 50 states and the District of Columbia. A total of 5,303 panelists were invited to participate, and 1,026 individuals completed the survey during the field period from May 6 to May 10, 2024, yielding a completion rate of 19.3% and a weighted cumulative response rate of 3.3%.

Participants were recruited from the Amplify AAPI Panel, a probability-based household panel designed to be representative of the U.S. AANHPI population. The panel includes members recruited through two sources: (1) a dedicated AAPI panel built using targeted address-based sampling and (2) AAPI respondents from NORC’s flagship AmeriSpeak Panel. Panel recruitment incorporated multistage sampling with stratification by age, gender, education, and geographic location. To enhance representativeness and reduce language-related selection bias, panel materials and surveys were available in English, Mandarin, Cantonese, Vietnamese, and Korean.

Surveys were administered using a mixed-mode design. Respondents completed the survey either online or via telephone interview, depending on their indicated preference during panel enrollment. Telephone interviews were conducted in English only. NORC implemented a randomized block design to rotate question order and mitigate order effects, as the Omnibus Survey included questions from multiple research sponsors.

To ensure data quality, NORC applied strict data cleaning procedures. A total of 103 cases were excluded due to response quality issues, including speeding, excessive item nonresponse, and straight-lining on grid items. Final analytic weights were computed in multiple stages: panel base weights, study-specific base weights accounting for sampling probabilities, and final weights adjusted for nonresponse and calibrated to U.S. Census American Community Survey (ACS) 2018–2022 benchmarks on age, gender, education, nativity, and AANHPI subgroup. Additional information about panel methodology and recruitment is publicly available [28].

### Measures

The survey instrument included a comprehensive set of sociodemographic measures developed and administered by NORC at the University of Chicago as part of the Amplify AAPI May 2024 Omnibus Survey. These measures were selected to capture key indicators relevant to social stratification, cultural background, and demographic heterogeneity within the AANHPI population.

#### Basic sociodemographic variables.

Respondents self-reported their *gender* (male, female), *age group* (18–29, 30–44, 45–59, 60+), and *educational attainment* (less than high school, high school diploma or GED, some college, bachelor’s degree, postgraduate/professional degree). *Annual household income* was categorized into five levels: less than $50,000; $50,000–$74,999; $75,000–$99,999; $100,000–$149,999; and $150,000 or more. *Marital status* was collected and classified as married, divorced/separated, or never married. *Employment status* was categorized as employed by others (employee), self-employed, not working (e.g., homemakers, job seekers), or retired. Respondents reported the *number of individuals residing in their household*, categorized as 1, 2, 3–4, or 5 or more persons.

#### Race/ethnicity.

Respondents were asked to identify their *specific AANHPI origin*, with mutually exclusive options including Chinese, Asian Indian, Filipino, Vietnamese, Korean, Japanese, Native Hawaiian/Pacific Islander, other singular AAPI origin, or multiple AAPI origins. Those who selected multiple categories were classified into a “multiple AAPI origins” category for analysis.

#### Nativity and generational status.

Participants were asked their place of birth and their parents’ place of birth, enabling classification into first generation (foreign-born), second generation (U.S.-born with foreign-born parents), or third-plus generation (U.S.-born with U.S.-born parents).

#### Language spoken at home.

Language spoken at home was assessed with a binary indicator: English or a non-English language (e.g., Mandarin, Cantonese, Korean, Vietnamese). The inclusion of Asian languages in survey administration aimed to reduce measurement error and underrepresentation of linguistically isolated households.

All sociodemographic variables were used in descriptive analyses and served as covariates or inputs to the latent class analysis (LCA), which aimed to identify meaningful subgroups within the AANHPI population based on shared patterns of educational attainment, income, language use, and generational status.

#### Ratings of perceived community well-being.

Two well-being ratings were included in the current analysis. First, for national well-being, participants rated, on a five-point scale, the well-being of “most people living in the United States” with response options ranging from “Excellent” to “Poor.” Second, for community well-being, participants rated the well-being of “the community in which you live” using the same five-point scale described above.

### Statistical analysis

The analytic plan was comprised of three steps: 1) identify latent classes based on key sociodemographic variables using the Amplified AAPI survey; 2) characterize the latent classes identified using additional variables; and 3) replicate the latent classes using a second sample. The analytic sample included 1,026 individuals with complete data on the variables used in the latent class analysis (LCA) and auxiliary characterization. LCA is a person-centered, model-based clustering technique used to identify unobserved (latent) subgroups within a population based on individuals’ patterns of responses across observed categorical variables. LCA was appropriate for this analysis because it enables the empirical identification of distinct subgroups of AAPI individuals who share similar sociodemographic profiles, without relying on arbitrary groupings. This method is particularly valuable for disaggregating heterogeneous populations to uncover meaningful subtypes that may differ in health, social, or behavioral outcomes.

LCA models were estimated in Mplus version 8.0 [29] using the manual three-step approach [30]. Four indicator variables were included in the LCA model: education level, annual household income, language spoken at home, and generational status in the U.S. The optimal class solution was determined by evaluating a combination of fit indices, including the negative two log likelihood (−2LL), Akaike Information Criterion (AIC), Bayesian Information Criterion (BIC), sample-size adjusted BIC (aBIC), the Vuong-Lo-Mendell-Rubin adjusted likelihood ratio test (VLMR), and the Lo-Mendell-Rubin (LMR) adjusted likelihood ratio test. For all log-likelihood-based indices (−2LL, AIC, BIC, aBIC), lower values indicate better model fit [31]. The VLMR and LMR tests provide significance tests comparing models with k versus k–1 classes (e.g., 4 versus 5 classes). We also considered the theoretical interpretability and sample sizes of the emergent classes.

To characterize the four latent classes, we examined associations with additional sociodemographic variables as well as the two well-being ratings not included in the class formation: age (categorized as 18–29, 30–44, 45–59, and 60+), Asian origin (nine categories), household size (1–6 + persons), marital status (married, divorced/separated, never married), and employment status (working for an employer, self-employed, not working, retired). These variables were treated as auxiliary variables in Mplus. The distal categorical outcome (DCAT) approach was used to compare proportions across latent classes without influencing class membership estimation, while the Bolck, Croon, and Hagenaars (BCH) method was used to examine relationships between latent class membership and continuous auxiliary variables.

For the two well-being ratings, chi-square tests were conducted to assess whether the distribution of these ratings differed significantly across the identified latent classes. All comparisons were conducted using listwise deletion for cases with missing auxiliary variable data (e.g., marital status), consistent with Mplus default settings. Significant omnibus and pairwise differences (*p* < .05) were used to interpret meaningful distinctions among the classes.

### Study 1 results

Table 1 presents the sample characteristics of the Amplify sample. This sample (N = 1,026) was nearly evenly split by gender, with 52.9% male and 47.1% female. The majority identified as Chinese (35.8%), and most participants were highly educated (84.8% held at least a bachelor’s degree), aged 30–59 (70.2%), and had household incomes of $100,000 or more (63.8%).

**Table 1:** Sample Characteristics of the Amplify Sample (*N* = 1,026).

Characteristics	Unweighted N	Unweighted %
Gender		
Male	543	52.9
Female	483	47.1
Asian origin^a^		
Chinese	367	35.8
Asian Indian	67	6.5
Filipino	94	9.2
Vietnamese	65	6.3
Korean	111	10.8
Japanese	102	9.9
Native Hawaiian/Pacific Island	21	2.0
Other singular AAPI origin	99	9.6
Multiple AAPI origins	100	9.7
Education		
Less than high school	10	1.0
High school	36	3.5
Some college	110	10.7
Bachelor’s degree	404	39.4
Post graduate/professional degree	466	45.4
Age, in years		
18–29	96	9.4
30–44	419	40.8
45–59	302	29.4
60+	209	20.4
Income, in dollars		
Less than 50,000	141	13.7
50,000 to 74,999	111	10.8
75,000 to 99,999	119	11.6
100,000 to 149,999	229	22.3
150,000 or more	426	41.5
Household size, in number of residents		
1	185	18.0
2	324	31.6
3 or 4	409	39.9
5 or more	108	10.5
Language spoken at home		
English	531	48.2
Other language	495	51.8
Generational status		
1^st^ (born outside U.S.)	452	44.1
2^nd^ (born in U.S. and parents born outside U.S.)	355	34.6
3^rd^ + (parents born in U.S.)	219	21.3
Marital Status		
Married/partnered	631	61.5
Divorced/separated	66	6.5
Widowed	14	1.4
Single, never married	315	30.7

*Note*. ^a^ Specific origin categories are exclusive. For example, respondents identifying as Chinese in this measure select no other AAPI origin.

### LCA Results

As shown in Table 2, we estimated latent class models with one through five classes using key sociodemographic variables (education, income, English language use at home, and generational status) to identify distinct subgroups within the AANHPI sample. Model selection was guided by a combination of statistical fit indices, likelihood ratio tests, and substantive interpretability.

**Table 2:** Model Fit Statistics and Likelihood Ratio Tests for Latent Class Analyses.

	−2LL	AIC	BIC	aBIC	VLMR	LMR	BLRT
1 Class	7714.466	7730.465	7769.933	7744.524	–	–	–
2 Classes	7399.754	7433.755	7517.623	7463.629	0	0	0
3 Classes	7269.626	7321.627	7449.896	7367.317	0.019	0.020	0
4 Classes	7227.902	7297.903	7470.573	7359.409	0.013	0.014	0
5 Classes	7213.526	7301.526	7518.597	7378.848	0.166	0.171	0.274

The four-class model was selected as the best-fitting and most interpretable solution. While the three-class model demonstrated strong fit, with improvements over the two-class model indicated by significant VLMR (*p* = .019) and LMR adjusted likelihood ratio tests (*p* = .020), the four-class solution provided additional model improvement. Specifically, the bootstrapped likelihood ratio test comparing the three- and four-class solutions was significant (*p* < .001), as were the VLMR (*p* = .0132) and LMR (*p* = .0142) tests. Although the four-class solution had a slightly higher BIC compared to the three-class model (7470.573 vs. 7449.896), the adjusted BIC favored the four-class model (7359.409 vs. 7367.317).

The average latent class probabilities for most likely class membership ranged from 0.749 to 0.913, indicating adequate classification certainty and separation among classes. Class proportions were as follows: Class 1 (30.6%), Class 2 (7.8%), Class 3 (17.7%), and Class 4 (43.9%). The four latent classes reflected distinct sociodemographic profiles. Class 1 was predominantly high-income, highly educated, English-speaking, and second- or third-generation individuals. Class 2 was a smaller group with lower income and education levels, predominantly English-speaking, and mostly third-generation. Class 3 included individuals with mixed educational attainment and income levels, primarily first-generation and limited English use at home. Class 4 was the largest group, characterized by high income, postgraduate education, and a high proportion of first-generation immigrants with limited English use at home.

### Class characterization results

**Fig 1:**
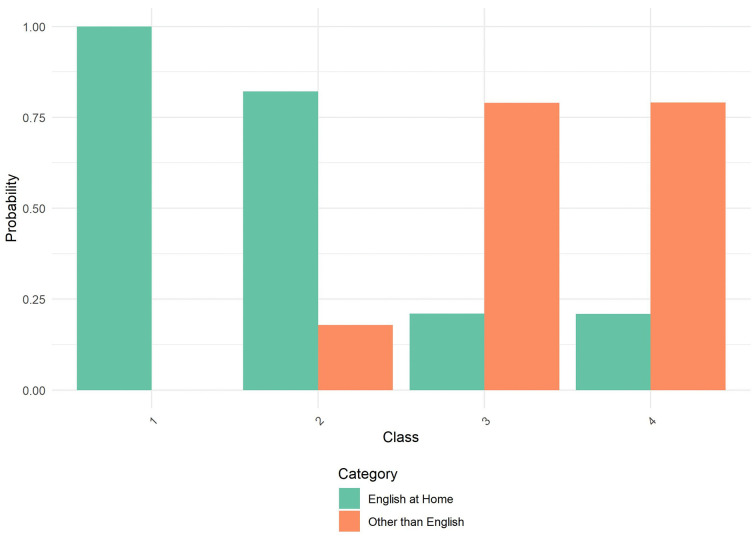
Probability by Class and Category for English at Home. Amplify Sample: Probability distribution of language spoken at home across the four latent classes identified in the latent class analysis. Classes 1 and 2 are predominantly composed of individuals who speak English at home. Classes 3 and 4 show substantially higher probability of speaking languages other than English at home. Categories include: English at Home (green) and Other than English (orange).

**Fig 2:**
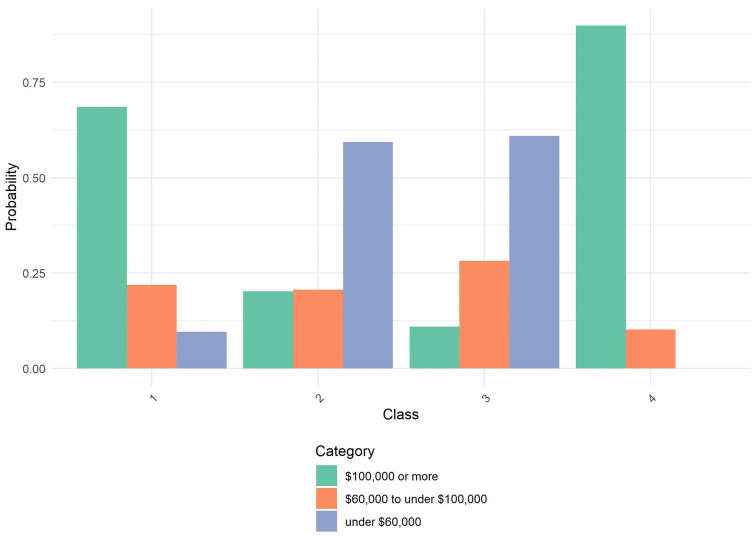
Probability by Class and Category for Income. Amplify Sample: Probability distribution of annual household income across the four latent classes identified in the latent class analysis. Class 1 is predominantly concentrated in the highest income bracket of $100,000 or more. Class 2 shows elevated probability in the under $60,000 income bracket. Class 3 demonstrates a more balanced distribution across income categories. Class 4 is concentrated in the highest income bracket. Categories include: $100,000 or more (green), $60,000 to under $100,000 (orange), and under $60,000 (blue).

**Fig 3:**
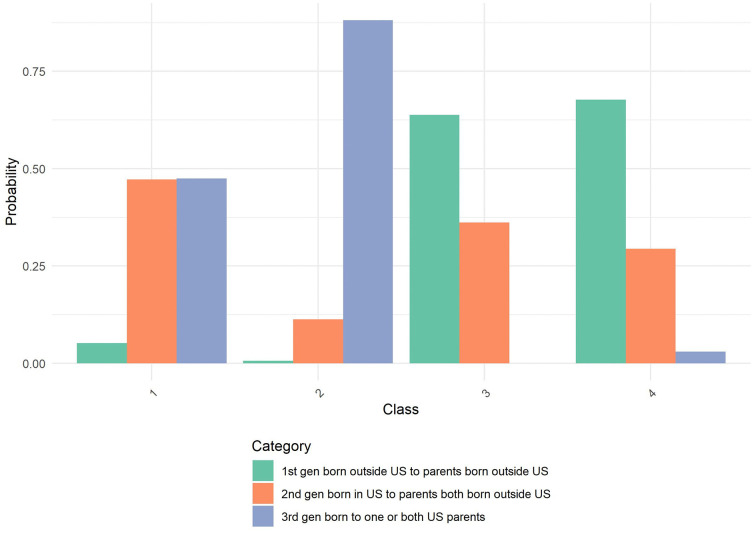
Probability by Class and Category for U.S. Generation. Amplify Sample: Probability distribution of generational and immigration status across the four latent classes identified in the latent class analysis. Class 1 is predominantly composed of individuals who are 2nd generation born in the US or 3^rd^ generation. Class 2 is heavily represented by 3^rd^ generation individuals born to one or both US parents. Class 3 shows substantial representation across all generational categories. Class 4 demonstrates high probability of being 1^st^ generation born outside the US to parents born outside the US. Categories include: 1st generation born outside US to parents born outside US (green), 2nd generation born in US to parents both born outside US (orange), and 3rd generation born to one or both US parents (blue).

**Fig 4:**
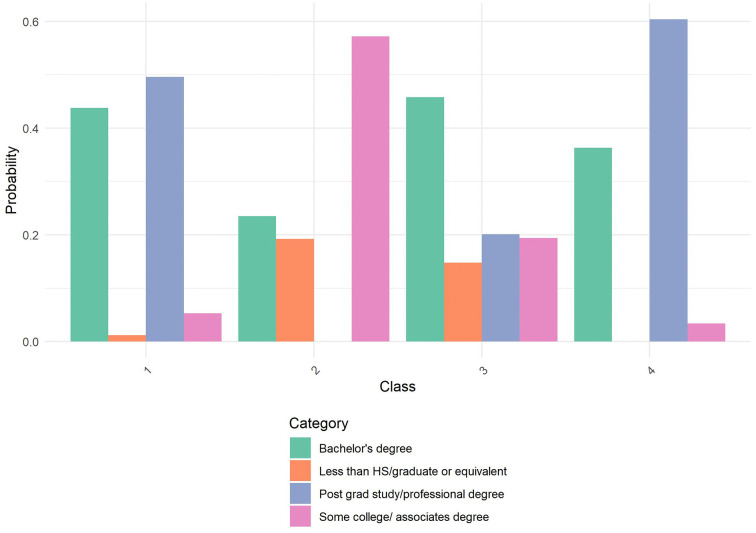
Probability by Class and Category for Education. Amplify Sample: Probability distribution of educational attainment levels across the four latent classes identified in the latent class analysis. Class 1 shows elevated probability of post-graduate or professional degrees. Class 2 demonstrates minimal representation in post-graduate education with higher representation in lower education categories. Class 3 shows a balanced distribution across multiple education levels. Class 4 is predominantly represented in postgraduate or professional degrees. Categories include: Bachelor’s degree (green), Less than high school/graduate equivalent (orange), Post-graduate study/professional degree (blue), and Some college/associate’s degree (pink).

After identifying the optimal four-class solution, we further characterized the latent classes using additional sociodemographic variables: age, Asian origin, household size, marital status, and employment status. Table 3 presents the class characterization results. Equality tests revealed significant differences across classes for all variables (p < .001), indicating meaningful heterogeneity in demographic composition. Figs 1–4 through 4 show the probability by class for each of these auxiliary variables.

**Table 3:** Class Characterization Results from the Amplify Sample.

	Class 1	Class 2	Class 3	Class 4
	Probability (SE)	Probability (SE)	Probability (SE)	Probability (SE)
**Age**				
18-29	0.045 (0.024)	0.134 (0.059)	0.274 (0.069)	0.055 (0.025)
30-44	0.392 (0.033)	0.284 (0.090)	0.259 (0.058)	0.507 (0.034)
45-59	0.307 (0.050)	0.171 (0.051)	0.203 (0.049)	0.346 (0.038)
60+	0.257 (0.047)	0.411 (0.075)	0.264 (0.051)	0.093 (0.017)
**AANHPI Origin**				
Chinese	0.337 (0.053)	0.003 (0.043)	0.300 (0.049)	0.453 (0.072)
Asian Indian	0.029 (0.014)	0.032 (0.036)	0.039 (0.026)	0.104 (0.026)
Filipino	0.135 (0.026)	0.094 (0.048)	0.143 (0.037)	0.043 (0.038)
Vietnamese	0.015 (0.020)	0.107 (0.055)	0.127 (0.056)	0.060 (0.016)
Korean	0.077 (0.022)	0.048 (0.030)	0.123 (0.048)	0.131 (0.025)
Japanese	0.220 (0.041)	0.323 (0.086)	0.020 (0.034)	0.020 (0.015)
Native Hawaiian/Pacific Islander	0.022 (0.019)	0.151 (0.056)	0 (0)	0.006 (0.006)
Other Singular AAPI Origin	0.004 (0.010)	0.056 (0.038)	0.188 (0.089)	0.123 (0.020)
Multiple AAPI Origins	0.160 (0.030)	0.185 (0.077)	0.059 (0.138)	0.060 (0.053)
**Household Size**				
1	0.151 (0.033)	0.49 (0.065)	0.175 (0.039)	0.110 (0.019)
2	0.410 (0.033)	0.289 (0.047)	0.259 (0.041)	0.278 (0.026)
3	0.197 (0.026)	0.103 (0.04)	0.201 (0.043)	0.233 (0.023)
4	0.166 (0.026)	0.044 (0.027)	0.192 (0.037)	0.270 (0.024)
5	0.052 (0.014)	0.053 (0.023)	0.080 (0.025)	0.068 (0.014)
6 or more	0.024 (0.010)	0.021 (0.016)	0.094 (0.029)	0.041 (0.012)
**Marital Status**				
Married	0.779 (0.038)	0.286 (0.071)	0.423 (0.079)	0.739 (0.067)
Divorced	0.058 (0.016)	0.128 (0.041)	0.115 (0.029)	0.015 (0.013)
Never Married	0.163 (0.035)	0.587 (0.090)	0.462 (0.091)	0.246 (0.059)
**Employment Status**				
Employee	0.649 (0.037)	0.181 (0.072)	0.378 (0.069)	0.805 (0.027)
Self-Employed	0.071 (0.018)	0.165 (0.070)	0.153 (0.035)	0.051 (0.014)
Not Working	0.070 (0.023)	0.370 (0.089)	0.290 (0.058)	0.091 (0.017)
Retired	0.210 (0.028)	0.284 (0.082)	0.179 (0.038)	0.053 (0.016)

**Class 1** (30.6%) consisted primarily of older individuals, with nearly 26% aged 60 or older and another 31% aged 45–59. Members of this class were highly educated, had high income, and almost exclusively spoke English at home. They were predominantly married (77.9%) and employed as salaried workers (64.9%). Most lived in two-person households. Asian origin composition was largely Chinese (33.7%) and Japanese (22.0%), with smaller proportions from other subgroups.

**Class 2** (7.8%) was the smallest class, characterized by older individuals (41.1% were age 60+), lower income, and lower educational attainment. This class had the highest proportion of one-person households (49.0%) and showed a diverse AAPI origin composition, including high representation of Japanese (32.3%) and individuals identifying with multiple AAPI origins (18.5%). Marital status was diverse, with only 28.6% married and a majority never married or divorced/separated. Employment status was also varied, with nearly 28.4% retired and 37.0% not working.

**Class 3** (17.7%) was composed of younger individuals, with 27.4% aged 18–29 and 25.9% aged 30–44. Members were predominantly 1st generation immigrants who spoke a language other than English at home. Educational attainment centered around a bachelor’s degree, and household sizes were relatively balanced. Class 3 had higher representation of Chinese (30.0%) and Filipino (14.3%) origin. Marital and employment status were also balanced, with 42.3% married and 37.8% employed, while 29.0% were not working.

**Class 4** (43.9%), the largest class, included younger adults primarily in the 30–44 (50.7%) and 45–59 (34.6%) age ranges. This group was highly educated, had high income, and was predominantly 1st generation, although language use at home varied. Members were largely of Chinese origin (45.3%), with others identifying across a range of AAPI backgrounds. Most were married (73.9%), lived in moderately sized households, and were working as employees (80.5%).

### Well-being ratings by latent class

We examined differences across the four latent classes in self-rated perceptions of national and community well-being using continuous ratings (1 = Excellent to 5 = Poor). Perceptions of national well-being were most negative in Class 2 (*M* = 3.745, *SE* = 0.120), followed by Class 3 (*M* = 3.465, *SE* = 0.072), Class 1 (*M* = 3.299, *SE* = 0.052), and Class 4 (*M* = 3.313, *SE* = 0.043), with relatively modest variation. These differences were statistically significant overall (*χ*² = 14.083, *p* = 0.003), with Class 2 differing significantly from Classes 1 and 4. Stronger variation was observed in perceptions of community well-being. Respondents in Class 2 rated their community’s well-being least favorably (*M* = 3.515, *SE* = 0.144), followed by Class 3 (*M* = 3.141, *SE *= 0.081), Class 4 (*M* = 2.640, *SE* = 0.049), and Class 1 (*M* = 2.549, *SE* = 0.060). These differences were also statistically significant (*χ*² = 62.457, *p* < .001), with multiple significant pairwise contrasts, especially between Class 2 and the other classes.

## Study 2: Analysis of the National Survey of Health Attitudes (NSHA) sample

### Methods

#### Participants and procedures.

Participants for the NSHA analysis were drawn from the 2023 National Survey of Health Attitudes (NSHA), a large, nationally representative survey designed to assess public views about health, well-being, and health equity across diverse U.S. populations. Data were collected by RAND in partnership with the Robert Wood Johnson Foundation. The final analytic sample (N = 5,620) combined two national probability-based online survey panels: the American Life Panel (ALP; n = 1,570) and the KnowledgePanel (n = 4,050), both of which are long-standing survey platforms commonly used for U.S. health and social research.

All ALP respondents included in the NSHA sample were panelists who had previously participated in the 2015 wave of the NSHA and remained active in the ALP at the time of the 2023 fielding. KnowledgePanel respondents were newly sampled for the 2023 administration, which allowed for adjustments to the sampling strategy to better meet project goals. Specifically, KnowledgePanel recruitment intentionally oversampled Black, Hispanic, and, where feasible, Asian American, Native Hawaiian, and Pacific Islander (AANHPI) respondents to support subgroup analyses. Sampling procedures were designed to ensure national representativeness and were weighted to reflect the broader U.S. adult population using post-stratification benchmarks from the American Community Survey.

Survey data were collected between March and April 2023 via self-administered web-based questionnaires. Respondents could complete the survey using either desktop or mobile devices. Participants provided informed consent electronically prior to participation, and survey instructions and items were available in English only. The full survey instrument included questions about perceived health, well-being, personal and community experiences with structural barriers, and attitudes toward equity-promoting policy actions. Data were accessed May-June 2025; authors had no access to information that could identify individual participants during or after data collection.

### Measures

#### Race/ethnicity.

To capture racial and ethnic diversity, survey participants were first asked to select all applicable racial and ethnic groups (e.g., Asian, Black or African American, Hispanic or Latino) and then provide further detail through subgroup categories. For instance, those selecting “Asian” could choose one or more detailed subgroups (e.g., Chinese, Vietnamese, Filipino, Korean, Indian, Japanese). This race/ethnicity item design follows the revised federal recommendations for data collection (OMB Statistical Policy Directive No. 15) and enables granular identification of ethnic subgroup identity [7]. Respondents identifying with Native American groups were asked to specify their tribal affiliation via free-text entry. Notably, participants could endorse more than one racial or ethnic identity, and as a result, some were categorized into multiple subgroups in descriptive analyses.

#### Well-being ratings.

Two well-being ratings were included in the current analysis as in Study 1. First, for national well-being, participants rated, on a five-point scale, the well-being of “most people living in the United States” with response options ranging from “Excellent” to “Poor.” Second, for community well-being, participants rated the well-being of “the community in which you live” using the same five-point scale described above.

### Statistical analysis

Similar to Study 1, we used LCA to identify distinct subgroups among Asian American respondents in the 2023 NSHA sample, employing a person-centered analytic approach. Given the relatively small number of AAPI respondents in the NSHA (*n* = 318), we focused on a reduced set of structural socioeconomic indicators to maximize interpretability and class stability while illustrating the utility of LCA in typical survey samples.

The final model included three categorical input variables: educational attainment (less than high school, high school diploma/GED, some college, bachelor’s degree or higher), annual household income (categorized into three levels: < $50,000; $50,000–$99,999; ≥ $100,000), and employment status (employed, unemployed, retired/other). Additional covariates, including census region, urbanicity, and homeownership, were considered in preliminary models but ultimately excluded from the final LCA due to insufficient variation and minimal contribution to class differentiation. The LCA method was detailed in the Study 1 Statistical Analysis section.

### Study 2 results

As shown in Table 4, the NSHA subsample (*N* = 318) was nearly evenly split by gender and predominantly comprised individuals of Chinese (29.9%), Asian Indian (17.0%), and Filipino (12.6%) origin. Most participants were highly educated (76.1% with a bachelor’s degree or higher), aged 30 and older (88.7%), and reported household incomes of $100,000 or more (66.4%).

**Table 4:** Sample Characteristics for NSHA Subsample (N = 318).

Characteristics	Unweighted N	Unweighted %
Gender		
Male	162	50.9
Female	156	49.1
Asian origin		
Chinese	95	29.9
Asian Indian	54	17.0
Filipino	40	12.6
Vietnamese	14	4.4
Korean	20	6.3
Japanese	24	7.5
Native Hawaiian/Pacific Island	14	4.4
Other singular AAPI origin	27	8.5
Multiple AAPI origins	14	4.4
None specified/missing	16	5.0
AANHPI and any other race/ethnicity*	9	2.8
Education		
Less than high school	8	2.5
High school	31	9.7
Some college	37	11.6
Bachelor’s degree	129	40.6
Post graduate/professional degree	113	35.5
Age, in years		
18–29	36	11.3
30–44	105	33.0
45–59	89	28.0
60+	88	27.7
Income, in dollars		
Less than 50,000	42	13.2
50,000 to 74,999	30	9.4
75,000 to 99,999	34	10.7
100,000 or more	211	66.4
Missing	1	0.3
Household size, in number of residents		
1	37	11.6
2	98	30.8
3	70	22.0
4	70	22.0
5 or more	43	13.6
Marital status		
Married/partnered	219	68.9
Divorced/separated	17	5.3
Widowed	3	0.9
Single, never married	79	24.8

Note. * Respondents in the “AANHPI and any other race/ethnicity” group (2.8%) were also included in other rows.

### LCA results

LCA was used to identify distinct sociodemographic subgroups among Asian American respondents in the 2023 NSHA sample (*N* = 318). The model incorporated educational attainment, household income, and employment status as indicator variables. Model fit statistics are presented in Table 5. The two-class solution was selected as the best-fitting model, demonstrating significantly improved fit over the one-class model across all indices (AIC, BIC, aBIC) and supported by statistically significant VLMR, LMR, and BLRT tests (*p* < .0001). The three-class solution did not yield significant improvement in model fit and was therefore not retained.

**Table 5:** Model Fit Comparison (NHSA Sample; N = 318).

	−2LL	AIC	BIC	aBIC	VLMR	LMR	BLRT
1 Class	1720.192	1732.192	1754.764	1735.734	–	–	–
2 Classes	1619.83	1645.830	1694.737	1653.503	<0.0001	<0.0001	<0.0001
3 Classes	1614.99	1654.990	1730.231	1666.795	0.3117	0.3242	0.7050

**Class 1 (High SES)**, which comprised 57.7% of the sample, was characterized by higher levels of educational attainment and household income, with a greater proportion of individuals currently employed. This group included more respondents in younger and middle-aged categories, with over one-third (38.9%) between the ages of 30 and 44. The majority of individuals in this class reported being married (73.4%) and owning their home (81.6%). In contrast, **Class 2 (Low SES)**, which comprised the remaining 42.3% of the sample, had lower income and education levels, and a higher representation of older adults, with 46.2% aged 60 and above. Members of this group were more likely to be retired or unemployed, renters (54.5%), and never married (32.8%).

Differences also emerged by census region and urbanicity, with Class 1 more likely to reside in the Northeast and Class 2 more likely to reside in the West. However, nearly all participants across both classes lived in urban areas, limiting the utility of rural-urban stratification.

### Class characterization results

**Fig 5:**
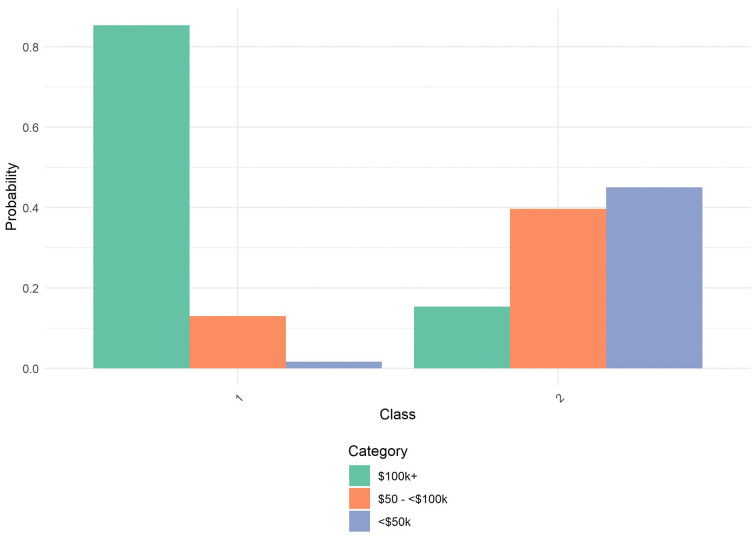
Probability by Class and Category for Income. NSHA Sample: Probability distribution of annual household income across the two latent classes identified in the latent class analysis. Class 1 is predominantly concentrated in the highest income bracket, reflecting high socioeconomic status. Class 2 is heavily concentrated in lower income brackets, with minimal representation in the highest income category, reflecting economic disadvantage. Categories include: $100,000+ (green), $50,000 - $100,000 (orange), and <$50,000 (blue).

**Fig 6:**
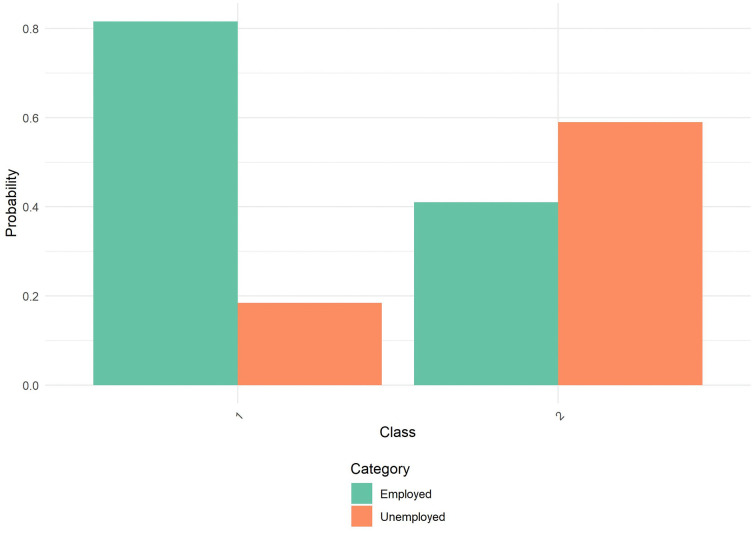
Probability by Class and Category for Employment Status. NSHA Sample: Probability distribution of employment status across the two latent classes identified in the latent class analysis. Class 1 demonstrates substantially higher employment probability, reflecting greater economic stability. Class 2 shows greater economic precarity, with substantially higher unemployment probability compared to Class 1. Categories include: Employed (green) and Unemployed (orange).

**Fig 7:**
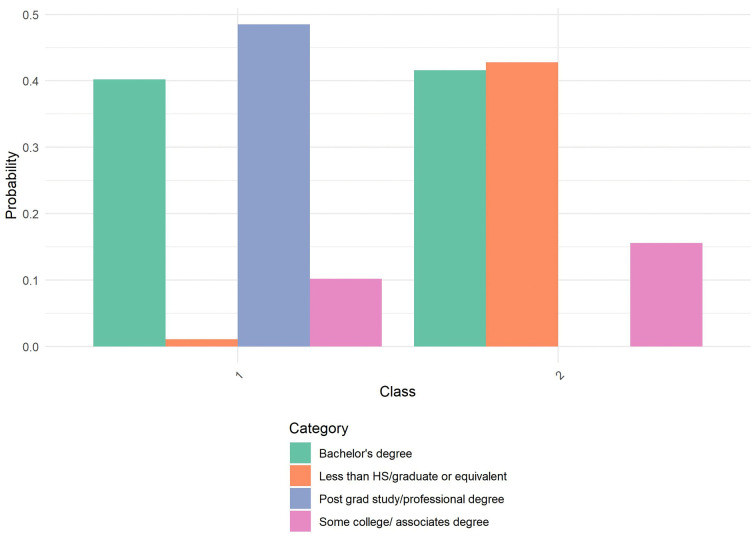
Probability by Class and Category for Education. NSHA Sample: Probability distribution of educational attainment levels across the two latent classes identified in the latent class analysis. Class 1 shows elevated probability of post-graduate or professional degrees, with minimal representation in lower education categories. Class 2 demonstrates a more balanced distribution across bachelor’s degrees, with virtually no representation in post-graduate education. Categories include: Bachelor’s degree (green), Less than high school/graduate equivalent (orange), Post-graduate study/professional degree (blue), and Some college/associate’s degree (pink).

Following the identification of the optimal two-class solution, we further examined demographic and structural differences between the classes using age, household size, marital status, homeownership, and geographic region. Class characterization results are presented in Table 6. Figs 5–7 illustrate the probability by class for each of these auxiliary variables.

**Table 6:** Class Characterization Results from the NSHA Subsample.

Characteristics	Class 1 (High SES)	Class 2 (Low SES)
**Age**		
18-29	0.094 (0.021)	0.166 (0.047)
30-44	0.389 (0.035)	0.167 (0.055)
45-59	0.307 (0.033)	0.205 (0.060)
60+	0.210 (0.032)	0.462 (0.080)
**Household ownership**		
Own	0.816 (0.026)	0.455 (0.070)
Rent	0.184 (0.026)	0.545 (0.070)
**Household size**		
1	0.119 (0.026)	0.110 (0.050)
2	0.275 (0.032)	0.394 (0.062)
3	0.259 (0.031)	0.118 (0.049)
4	0.232 (0.030)	0.190 (0.049)
5	0.066 (0.018)	0.110 (0.044)
6 or more	0.049 (0.026)	0.077 (0.055)
**Marital status**		
Married	0.734 (0.031)	0.584 (0.067)
Divorced/Separated	0.042 (0.014)	0.088 (0.035)
Never Married	0.224 (0.030)	0.328 (0.062)
**Urbanicity**		
Urban	0.981 (0.010)	0.936 (0.028)
Rural	0.019 (0.010)	0.064 (0.028)
**Region**		
Northeast	0.266 (0.030)	0.139 (0.047)
Midwest	0.073 (0.018)	0.120 (0.040)
South	0.197 (0.027)	0.155 (0.046)
West	0.465 (0.034)	0.587 (0.063)

**Class 1 (57.7%)** represented a high socioeconomic status profile, characterized by younger and middle-aged individuals: nearly 39% were aged 30–44 and 31% were aged 45–59, with only 9.4% under 30. This class had the highest homeownership rate (81.6%) and the lowest proportion of renters (18.4%). Most respondents lived in moderately sized households of two to four people. A strong majority were married (73.4%), and only 4.2% were divorced or separated. The group was overwhelmingly urban (98.1%) and regionally distributed across the West (46.5%), Northeast (26.6%), and South (19.7%), with fewer respondents in the Midwest (7.3%).

**Class 2 (42.3%)** reflected a lower SES profile, with nearly half (46.2%) of respondents aged 60 or older. This class had greater economic precarity, as reflected in the higher rate of rental housing (54.5%) and lower homeownership (45.5%). Household composition skewed smaller, with 39.4% living in two-person households and a slightly higher proportion reporting larger households (5 + members) compared to Class 1. Just 58.4% were married, while 32.8% were never married and 8.8% were divorced or separated. Although a majority resided in urban settings (93.6%), this group had the highest rural representation (6.4%). Regionally, respondents in this class were more concentrated in the West (58.7%) and had lower representation in the Northeast (13.9%).

### Well-being ratings by latent class

We examined differences across the two latent classes in self-rated perceptions of national and community well-being, rated on a scale from 1 (Excellent) to 5 (Poor). Mean ratings of national well-being did not significantly differ across classes (High SES: *M* = 3.41, *SE* = 0.06; Low SES: *M* = 3.28, *SE* = 0.10; *χ*² = 1.098, *p* = 0.295). However, a significant difference emerged for community well-being (*χ*² = 5.689, *p* = 0.017), with the High SES class reporting a more favorable mean rating (*M* = 2.35, *SE *= 0.06) compared to the Low SES class (*M* = 2.66, *SE* = 0.11).

## General discussion

Building from an effort in the 2023 NSHA to collect additional data that would better reflect health attitudes within the AANHPI community, this exploratory study aimed to utilize LCA to disaggregate data from AANHPI community members into meaningful subgroups to inform practice. Findings from this study underscore the demographic complexity and heterogeneity within the AANHPI population. The LCA analysis identified four latent classes in the larger Amplify sample and two latent classes in the smaller NSHA sample. These classes represent distinct subgroups that vary not only in socioeconomic characteristics but also in language use, generational status, and national origin.

When compared to disaggregating by AANHPI origin group alone, the LCA analysis provided an opportunity for more nuanced insights, inclusive of several important social determinants of health, overcoming sample size limitations. The LCA results indicate that even in a relatively small and traditionally sampled survey like the NSHA, latent class analysis can reveal meaningful sociodemographic heterogeneity within aggregated racial/ethnic categories. Class characterization results suggest that meaningful class-based stratification exists within the AANHPI NSHA subsample. While both classes were predominantly urban due to the nature of the respondent pool, substantial differences emerged in age structure, household stability, marital status, and homeownership—factors that likely influence perceptions of well-being and opportunity. The two-class solution captures key structural dimensions of heterogeneity that may be obscured in aggregate racial/ethnic data.

Given the staggering cost of health inequities [1] and limited resources available for public health entities to address these health inequities, it is crucial that data disaggregation efforts be feasible and informative for action and practice [32]. While origin-specific factors such as culture, language, indigeneity, and history are important for developing tailored community-humble supports, origin-focused disaggregation approaches are limited by the availability of data on origin [14]. Given current societal shifts that may limit the collection and use of data, such as race or ethnicity, to identify potential health disparities, it is essential that public health efforts continue to utilize available data to inform action [2].

Community based organizations and public health researchers have typically had limited capacity to utilize methods such as LCA in their research due to limited access to appropriate training and software. However, public health training programs offer programs in these methodologies, and some public health departments are training their own staff in utilizing these methods to address health inequities within their communities. Additionally, freely-available packages developed for use in the R environment can conduct LCA, allowing for typically resource-constrained research teams to use these methods [33–35]. As more public health researchers adopt R as a standard programming language, the feasibility of incorporating methods such as LCA may continue to improve.

This study utilized observable variables associated with health inequities, such as educational attainment [36], household income [37], language spoken at home [38], and generational status [39], to identify latent groups within the AANHPI populations sampled. Both LCA and clustering methods have been used in intersectionality research to identify subgroups within populations often treated as homogenous and have the potential to inform more tailored public health practice [26]. The identified differences in community well-being underscore how these sociocultural factors may shape individuals’ local outlooks and perceptions of health-related opportunity, in ways not readily captured by ancestry or racial/ethnic labels alone. These differences emphasize the limitations of treating AANHPI individuals as a single, uniform group in research or practice. Using these statistical approaches to identify potential inequities within an aggregated racial group may also allow for investigations that assess the impact of racism, a health risk factor that is often assessed for by proxy using variables collected for race or ethnicity [18]. While it remains important to consider origin-specific factors when tailoring interventions and practices for AANHPI communities, reviewing the distribution of respondent AANHPI origin across classes and well-being indicators illuminates the potential opportunities of a person-centered disaggregation approach. LCA is not intended to replace disaggregated data collection and analysis; rather, LCA is offered as a complementary strategy.

This study has several limitations. Although the included surveys were intended as representative samples, the unweighted data were used in this particular analysis, limiting the generalizability of the results. The majority of respondents for both the NSHA and Amplify panels endorsed being homeowners, living in urban areas, having a household income over $100,000, being married, and having a bachelor’s degree or higher; this may have resulted in an overrepresentation of individuals with higher socioeconomic status. Adequate representation of diverse AANHPI community members in health surveys and research is a longstanding issue [14,40]. While the LCA approach described in this study is an important tool, complementary community-engaged research is an important step to identify information on the health status and needs of community members underrepresented or excluded from existing data [41]. This research can also serve to explore community- and culturally-specific aspects of health that existing tools and instruments are unable to capture. As the Amplify panel did not capture information on Multiracial/ethnic AANHPI community members with racial/ethnic identities outside of the AANHPI categories, the LCA analyses for each of the studies excluded individuals who identify as AANHPI and some other race or ethnicity. Multiracial/ethnic AANHPI people are a growing part of the AANHPI community, particularly within younger generations [42]. Public health data continue to identify Multiracial/ethnic communities, including Multiracial/ethnic AANHPI community members, as experiencing a high burden of adverse health outcomes, such as suicidal thoughts and behaviors [42–44]. Future research should consider how to include Multiracial/ethnic AANHPI community members in research, and practice recommendations should be inclusively developed.

## Conclusion

What individual and societal differences, variations, and factors genuinely matter to health outcomes and health attitudes? This exploratory analysis suggests the benefits of a shift in disaggregation perspective, focusing on the relevance and impact of the identified variables rather than solely on the breadth of disaggregation. While this study used AANHPI populations as an illustrative example, LCA can be used within any population to disaggregate data and inform policy and practice recommendations.

This study demonstrates the utility of applying a person-centered, disaggregated analytic approach to capture the within-group heterogeneity of the AANHPI communities. The Amplify panel was intentionally designed to improve on standard national survey limitations. While an improvement, even dedicated sampling has limitations in capturing subpopulations. This study demonstrated the opportunity for methods such as LCA to elucidate critical insights on the health status and needs of AANHPI subgroups that may be masked by aggregated data and/or non-representative samples. This is crucial for informing tailored resource allocation and strategies that reflect the multifaceted circumstances of AANHPI individuals [45]. Critically, LCA can also support efforts to disaggregate data from a larger group using available variables. This process can inform advancements in research and practice and can support practitioners throughout the public health and healthcare system to consider other social factors that drive health inequities in different subpopulations. This approach can also serve to allow for the adoption of more inclusive analytic methods capable of capturing unmeasurable differences within the group.

It is important to recognize the value of intentional efforts to collect sufficient data to speak to the health needs within populations often underrepresented in health data when opportunities exist, as evidenced by the existence of the Amplify panel. Even if limited to origin-only stratification, the Amplify panel highlighted several differences across origin-groups in perceptions of well-being, particularly for people identifying as Native Hawaiian and Other Pacific Islander, that were not captured by the more traditionally sampled NSHA study. The sample size of the Amplify panel also allowed for more nuanced LCA, which can further inform future disaggregation efforts and the tailoring of supportive resources. Future use of LCA could be enhanced with targeted oversampling of smaller racial/ethnic communities. We encourage researchers to continue exploring how to better understand the health status and needs of populations underrepresented or excluded from existing data. Approaches such as integrating an oversample into an existing survey, conducting a complementary study that aims to generate a more representative sample, and partnering with communities to support the generation of data on communities by communities are examples of how we can bridge known and unknown data gaps.

We must also recognize the ongoing shortage of resources for public health activities and the increasing cost of healthcare, which will likely result in increased competition for available resources. Simultaneously, the availability of data on social drivers of health inequities within existing and future datasets is uncertain, requiring researchers to consider different avenues to inform practice recommendations. This study presents a feasible approach to data disaggregation using variables that were available in each study and provides important insights on differences in outcome variables that are important for practitioners to consider as resources are allocated. When paired with staff training in LCA methodologies, this approach may help health agencies make more efficient use of these limited resources. Future research and policy should continue to embrace these methodologies to promote data-driven solutions for decision-making that go beyond the data aggregate to make meaningful progress toward reducing differences in health outcomes.
